# Searching for Novel Air Pollutants Inducers of Toxicity in the Respiratory and Immune Systems

**DOI:** 10.3390/toxics10040149

**Published:** 2022-03-22

**Authors:** Irene Camacho

**Affiliations:** Faculty of Life Sciences, Campus Universitário da Penteada, Madeira University, 9020-105 Funchal, Portugal; ireneg@staff.uma.pt

Many contaminants may pollute the indoor or outdoor environment in a variety of ways. Almost any toxic chemical, physical or biological agent could make its way into the atmosphere to pollute the air that we breathe. The World Health Organization (WHO) data shows that almost the entirety of the global population breathes air that exceeds WHO guideline limits [1], containing high levels of pollutants, with low- and middle-income countries suffering from the highest exposures. According to that organization, the burden of disease attributable to air pollution is now estimated to be on a par with other major global health risks, such as unhealthy diet and tobacco smoking, being currently recognized as the biggest environmental threat to human health.

The interactions between the human body and air pollutants have been extensively studied. Air pollution has caused high rates of morbidity and mortality in the human population, having a great impact on quality of life. Urban ambient air pollution consists of gaseous components and particulate matter (PM), the latter being analysed in detail over the last decades. 

Inhalation and deposition of PM in the respiratory system can cause allergic or toxic responses. The deposition of these particles in the respiratory tract depends on particle properties such as size shape, density, airway morphology and breathing pattern. Particles with a size > 10 µm are normally filtered by the upper airways. Coarse particles or PM_10_ (≤10 μm in diameter), fine particles also called PM_2.5_ (≤2.5 μm in diameter) and ultrafine particles (UFP), ≤0.1 μm in diameter, can reach the lower airways, with the smallest ones accumulating in the terminal bronchioles and alveoli and the PM_10_ mostly in the conducting airways. Coarse particles may include dust, smoke, bacteria, mould spores and airborne viral particles, while fine particles can come from natural or human-made sources, namely vehicle exhaust, wildfires, power plant emissions and other combustion activities. On the other hand, UFP are found in nature (e.g., from forest fires, viruses) or derive from anthropogenic sources (e.g., combustion processes, synthetic and engineered products) [2].

From a physio-toxicological perspective, when inhaled pollutants penetrate into the airways, they first contact the primary barrier to all environmental compounds, which is the bronchial epithelium. PM tends to interact with the respiratory epithelial cells, activating a signalling pathway and NLRP3 inflammasome, inducing several pro-inflammatory cytokines and granulocyte-macrophage colony-stimulating factors by innate immune cells like macrophages, neutrophils and dendritic cells. Innate immune cells, like alveolar macrophages, can also promote the clearance of particles via phagocytosis, along with the normal mucociliary transport. Both innate and adaptive pathways eventually lead to the induction of oxidative damage and lung injury [3].

There is evidence that UFP have the capacity to diffuse into the systemic circulation reaching as far as the heart, liver, spleen or brain. Particles can be eliminated through distinct pathways depending on the site of deposition. In addition, depending on the dose and the frequency, the distinct air pollutants might exhibit a particular toxicological mechanism. In the case of ozone, it can induce different injuries and inflammation in the lungs. It is already known that being exposed to even a small amount of ozone can cause an asthma exacerbation and further worsening of the symptoms of respiratory diseases, leading to an increase in mortality. Low levels of carbon dioxide combined with exposure to organic dust can alter immune responses leading to more airway inflammation and induction of pro-inflammatory cytokines. Additionally, an association between volatile organic compounds (VOCs) and pulmonary disease, including symptoms like wheezing and throat irritation, has been reported. VOCs encompass hundreds of chemicals that have been applied in a wide range of household and industrial products. In fact, human exposure to VOCs is pervasive and epidemiologic studies, especially in occupational settings, describe the association between VOC exposures and adverse health outcomes, primarily carcinogenicity and neurological effects [4,5]. Apparently, exposure to higher VOCs levels in human subjects in daily life is suggested to induce changes in airway inflammation and possibly increase T-helper Th2 inflammation [3,6].

Research involving both in vivo and in vitro experiments have allowed greater understanding of the effects of air pollution on human airways. The use of murine models to investigate specific features of allergic asthma, combined with exposure to particulate matter, showed that PM_2.5_ and PM_10_ exacerbated the allergic airway response. Likewise, epidemiological studies, controlled human exposure and ex vivo studies have been used to assess the human health impact of air pollution [3,7].

There are still limited data on the effects of multiple exposures to air on disease outcomes. Patients with chronic obstructive diseases such as asthma and COPD are especially vulnerable to the harmful effects of air pollution. However, the quantitative contribution of air pollution to these diseases is not exactly known in humans [8].

Large segments of the world population inhale many of the above-mentioned air pollutants on a daily basis. Further, there is limited information regarding new inhaled toxicants that the human body can be exposed to, particularly under the current geoclimatic and social context. Human population is facing unprecedented invisible threats [9], such as toxic gases and particles derived from natural sources (e.g., volcanic eruptions), conflict scenarios, migrations, or even biological particles from invasive plants of which respiratory and immunological effects are not well known. On the other hand, there are novel inhalable substances of technological origin of which toxicological effects in the respiratory and immune systems are unreported yet. 

The ability of emerging chemical pollutants to have harmful effects on human health and the environment is becoming evident, especially by the increasing trade of chemical products at a global scale. Ultimately, the new investigational trends capable of increasing the insight on underexplored air pollutants that are affecting human heath, for either indoor or outdoor environments, should be performed.
